# Outcomes of simultaneous resection for colorectal liver metastases: A nationwide cohort study (2005–2022)^☆^

**DOI:** 10.1016/j.sopen.2025.07.008

**Published:** 2025-08-05

**Authors:** J.H. Angelsen, S. Yaqub, T.A. Hegvik, L.S. Nymo, T. Veen, V.J. Dagenborg, E.A. Bringeland

**Affiliations:** a Department of Gastroenterological Surgery, Haukeland University Hospital, Bergen, Norway; b Institute of Clinical Medicine, Faculty of Medicine, University of Bergen, Norway; c Department of Hepato-Pancreato-Biliary Surgery, Oslo University Hospital, Oslo, Norway; d Institute of Clinical Medicine, University of Oslo, Oslo, Norway; e Department of Surgery, St. Olavs Hospital, Trondheim, Norway; f Department of Clinical and Molecular Medicine, Norwegian University of Science and Technology, NTNU, Trondheim, Norway; g Department of Circulation and Medical Imaging, Norwegian University of Science and Technology (NTNU), Trondheim, Norway; h Department of Global Public Health and Primary Care, University of Bergen, Bergen, Norway; i Department of Surgical Oncology, The Norwegian Radium Hospital, Oslo University Hospital, Oslo, Norway; j Department of Gastroenterological Surgery, University Hospital of North Norway, Tromsø, Norway; k Institute of Clinical Medicine, Arctic University of Norway, Tromsø, Norway; l Department of Gastrointestinal surgery, Stavanger University Hospital, Stavanger, Norway

**Keywords:** Colorectal cancer, Synchronous colorectal liver metastases, Simultaneous resections, Overall survival, Complications, Major liver resctions

## Abstract

**Introduction:**

The optimal treatment strategy for synchronous colorectal liver metastases (CRLM) has been a topic for ongoing debate. We present a national cohort containing simultaneous resections of the primary tumour and CRLM, with emphasis on postoperative complications, and survival.

**Method:**

This population-based national cohort study included consecutive patients who underwent simultaneous resections in Norway between 2005 and 2022. Postoperative complications (Accordion Severity Grading System – ASGS) and overall survival (OS) were analysed in relation to the extent of liver resection and location of the primary tumour.

**Results:**

A total of 193 patients underwent simultaneous resection of the primary tumour and liver metastases. Postoperative complications graded as ASGS ≥3 occurred in 48 (24.9 %) while 23 (11.9 %) patients developed ASGS ≥4 complications. Anastomotic leakage was observed in 9 (4.7 %) patients. Among 18 (9.3 %) patients undergoing major liver resections, 10 (55.6 %) and seven (38.9 %) patients experienced ASGS ≥3 and ASGS ≥4 complications, respectively. Major liver resection was an independent predictor for ASGS ≥3, (RR: 2.40; p = 0.002). The 30- and 90-day mortality rates were 0.5 % (n = 1) and 2.1 % (n = 4), respectively. Median, 5- and 10-year OS were 4.8 years, 46.7 % and 35.6 %, respectively. In a multivariable analysis both an increasing number of liver metastases and ASGS ≥4 complications were independently associated with inferior OS.

**Conclusions:**

Simultaneous resection for synchronous CRLM was a safe option, particularly in minor liver resections. Major liver resections were associated with increased risk of severe complications and inferior OS.

## Highlights

• This study present data from a comprehensive nationwide cohort of patients that underwent simultaneous resection for colorectal liver metastases.
• Postoperative complications (Accordion ≥3) occurred in 1 of 4 patients. Anastomotic leakage was observed in 4.7 % of the patients.
• Major liver resection (≥ 3 segments) was an independent predictor for Accordion ≥ 3 complications.
• Increasing number of liver metastases and severe complications (Accordion ≥ 4) were independently associated with inferior overall survival
• Median, 5- and 10-year overall survival were 4.8 years, 46.7% and 35.6%, respectively

## Introduction

The optimal treatment strategy for patients with colorectal cancer (CRC) and synchronous liver metastases (CRLM) has been debated for decades. Conflicting evidence exists regarding the outcomes of simultaneous versus staged resections, particularly in terms of postoperative morbidity and overall survival (OS) [[1], [2], [3]]. The advocated advantage of the simultaneous approach is the potential to reduce the total number of surgical interventions and expedite complete tumour clearance, which may lead to health-economic benefits, shorter time to functional recovery, reduced psychological stress [[4], [5], [6], [7]], and possibly improved oncological outcomes. However, concerns persist that combining two major surgical procedures may lead to an unacceptably high risk of postoperative morbidity and mortality [8]. This is especially relevant in patients with locally advanced rectal cancer who undergo neoadjuvant chemoradiotherapy, for whom a staged approach has traditionally been favoured to reduce the risk of complications [9]. Nevertheless, additional preoperative considerations such as patient age, comorbidities, frailty, polypharmacy, smoking status, tumour stage, and the number and size of liver metastases may impact the risk of severe complications and reduced long-term survival [10]. This study aims to investigate whether the location of the primary tumour and the extent of liver resection were associated with complication rates and long-term survival in patients undergoing simultaneous resection of the CRC and liver metastases, using data from a national cohort of consecutive patients.

## Methods

This study constitutes a nationwide, population-based retrospective cohort analysis of patients who underwent simultaneous resection for their primary CRC and synchronous CRLM between 2005 and 2022. Data were collected from all five hepatopancreatobiliary (HPB) surgical centres in Norway, covering a catchment area of approximately 5.5 million people. Clinical data were retrieved from the patients' medical records. OS was counted from date of surgery to death of any cause. The administrative censoring date was June 15th, 2024.

### Preoperative assessments

All cases were evaluated at multidisciplinary tumour board meetings. The national clinical guidelines supported simultaneous resections primarily in patients eligible for minor liver resections and colorectal surgery. Simultaneous procedures involving hemi-hepatectomies were performed only in highly selected cases, predominantly for patients with the primary tumour located in the colon. Eligibility criteria included good performance status (Eastern Co-operative Oncology Group (ECOG) 0–1), and minimal comorbidities. Patients were scored according to the American Society of Anaesthesiologists (ASA) physical status classification. Liver resection was considered appropriate if ≥30 % of the future liver remnant was tumour free in at least two adjacent segments with preserved blood perfusion and biliary drainage, or ≥40 % when subjected to preoperative chemotherapy.

During the study period, surgical indications for patients with oligometastatic disease have gradually expanded. Initially, the presence of pulmonary or peritoneal metastases was considered a contraindication to liver surgery but was later included in cases with resectable extrahepatic disease. In the recent years, patients with indolent, non-resectable pulmonary metastases were accepted for liver resection based on favourable prognoses [11]. In selected cases, patients undergoing cytoreductive surgery with hyperthermic intraperitoneal chemotherapy were also considered candidates for simultaneous liver resections [12].

### Chemotherapy and radiotherapy

Systemic chemotherapy was administered in either a neoadjuvant/perioperative, or in a downsizing context. In patients with limited extrahepatic disease and liver metastases, chemotherapy served as a test of biological responsiveness. Oxaliplatin-naïve patients deemed upfront resectable, received perioperative Nordic FLOX or FOLFOX (four-six cycles preoperatively and six-eight postoperatively) [[13], [14], [15]]. To maximize response in patients with potentially resectable CRLM, various systemic treatment regimens were administered based on individual tumour mutation profiles (KRAS, NRAS, BRAF) [16]. First line treatment with FOLFOX or FOLFIRI was commonly combined with EGFR-inhibitors (for wildtype tumours) or angiogenesis inhibitors such as bevacizumab (for mutated tumours). Locally advanced rectal cancers were managed with neoadjuvant radiotherapy (RT), guided by MRI-assessed circumferential resection margins ≤ of 1 mm. Of the patients that received RT, most had long-term RT (50 Gy in 25 fractions with concomitant 5-fluorouracil or capecitabine). In selected cases requiring integration with liver-directed therapy, short-course RT (5 × 5 Gy) was employed for logistical efficiency.

### Surgery

Colon cancer surgery adhered to the principles of D2/D3 lymphadenectomy with complete mesocolic excision. Rectal cancer surgery included low anterior resection or abdominoperineal resection with permanent stoma. Protective ileostomy was considered in patients with low anastomoses or preoperative RT. Liver resections were performed as parenchyma preserving procedures whenever feasible and were classified as either minor (wedge, segmentectomy, or bisegmentectomy) or major (resection of ≥ three adjacent segments). Resection margins <1 mm were classified as R1 [17]. Postoperative complications were classified according to the Accordion Severity Grading System (ASGS) [18].

### Follow-up

Follow-up included CT scans of the chest, abdomen and pelvis every 4–6 months up to five years postoperatively. The follow-up intervals varied somewhat between the different health-regions in Norway.

### Statistics

Descriptive statistics are presented as (n, %) or summarized by median (Interquartile range, IQR). The chi-square (*χ*^2^) test was used to calculate univariate associations between categorical variables, and the Mann-Whitney *U test* for continuous variables. Poisson regression with robust standard errors was used to calculate risk ratios (RR) and 95 % confidence intervals (CI) for the associations between various variables and the presence of complications [19]. This approach was chosen over logistic regression to avoid odds ratio inflation for common outcomes [[19], [20], [21], [22]].

Overall survival was analysed using Kaplan–Meier (K-M) estimates and differences tested for significance with the log-rank test [23,24]. A Cox proportional hazard regression model was used in a multivariable analysis of long-term survival rates [25]. A p-value of <0.05 was considered statistically significant. All analyses were performed using SPSS version 26 (IBM Corp., USA) and R version 4.3.3 (R Foundation for Statistical Computing, Austria).

### Ethical considerations

The study was approved by the Regional Committees for Medical and Health Research Ethics, Western region of Norway, 2023 (REC number: 198367) and Data Protection Officer (Haukeland University Hospital, PVO number: 4143).

## Results

A total of 193 patients underwent simultaneous resection for CRC and CRLM between 2005 and 2022 in Norway. The distribution of resections among the five hospitals performing liver resections varied according to their respective catchment areas (Supplemental Table 1).

Baseline data are presented in Table 1. Median age was 63.6 years (IQR 54.3–71.2), and 91 (47.2 %) patients were female. Preoperative chemotherapy was administrated to 96 (49.7 %) patients. The primary tumour was located in the colon in 130 (67.4 %) patients, and rectum in 63 (32.6 %) patients. Baseline extrahepatic metastases were identified in the peritoneum in 18 patients (9.3 %) and in the lungs in 16 patients (8.3 %). Rectal cancer was more frequent in males (n = 41 (40.2 %)) vs. females (n = 22 (24.2 %)), p = 0.018. Major liver resections (≥3 segments) were performed in 18 patients (9.3 %). Age distributions were similar between the minor (median 63.8 years) and the major (median 61.2 years) liver resection groups; p = 0.225, Table 1. Median duration of surgery was 260 min (IQR 202-330). Minimally invasive surgery (MIS) was performed in 37 (19.2 %) patients.

**Table 1:** Baseline characteristics of patients with colorectal cancer and synchronous liver metastases 2005–2022 undergoing simultaneous resection stratified by location of primary tumour, n = 193.

	Colon⁎, n (%) n = 130	Rectal, n (%) n = 63	Total, n (%) n = 193	p
Age⁎⁎⁎	64 (57.0–72.0)	61.3 (50.1–70.2)	63.6 (54.3–71.2)	0.073^4^
Gender				0.017^1^
Male	61 (46.9)	41 (65.1)	102 (52.8)	
Female	69 (53.1)	22 (34.9)	91 (47.2)	
Radiation preop	NA	32 (50.8)	32 (16.6)	
Chemo preop	59 (45.4)	37 (58.7)	96 (49.7)	0.082^1^
Perioperative	30 (23.1)	32 (50.8)	62 (32.1)	<0.001^1^
Downsizing⁎⁎	29 (22.3)	5 (7.9)	34 (17.6)	
Extrahep. mets.				0.187^1^
Peritoneum	15 (11,5)	3 (4.8)	18 (9.3)	
Lung	10 (7.7)	6 (9.5)	16 (8.3)	0.567^1^
No. of mets⁎⁎⁎	2 (1–3)	2 (1–3)	2 (1–3)	0.597^4^
ASA score				0.037^1^
1	4 (3.1)	4 (6.5)	8 (4.2)	
2	66 (50.4)	42 (66.7)	108 (56.8)	
3	52 (40.6)	16 (25.8)	68 (35.8)	
4	6 (4.7)	0 (0)	6 (3.2)	

1) Pearson Χ^2^, 2) Including left lateral resection, 3) Right or left hepatectomy, 4) non-parametric test (independent median samples test).

⁎ Distribution in the colon: coecum: n = 27 (14.0 %); ascending colon: n = 38 (19.7 %); transverse colon including the flexures: n = 16 (8.3 %); descending colon: n = 5 (2.6 %), and sigmoid colon: n = 44 (22.8 %).

⁎⁎ Downsizing: Chemotherapy was administered for stabilizing indication in 12 patients undergoing cytoreductive surgery included liver resection.

⁎⁎⁎ median (IQR).

### Postoperative complications and mortality

Severe complications (ASGS ≥3) were observed in 48 patients (24.9 %), while the remaining 145 (75.1 %) experienced no or mild/moderate complications (Table 2). Reoperations were performed in 14 patients (7.3 %), with anastomotic leakage (nine patients, 4.7 %) and bowel obstruction (two patients, 1 %) being the most common indications. Details from the 23 patients with ASGS ≥4 complications are further outlined in Supplemental Table 2. Among patients who underwent minor liver resections, 38 (21.7 %) experienced ASGS ≥3 complications, compared to 10 (55.6 %) following major liver resections (p = 0.002). Similarly, ASGS ≥4 complications were observed in 16 cases (9.1 %) after minor resections and seven (38.9 %) cases following major resections, p < 0.001. Major liver surgery was the only independent predictor for ASGS ≥3 complications (RR 2.40, (95 % CI: 1.37, 4.17), p = 0.002, Table 3). ASA scores of 3 and 4 were significantly more frequent in colon cancer (Table 1), but ASA score was not independently associated with increased complication rates (Table 3).

**Fig. 1:**
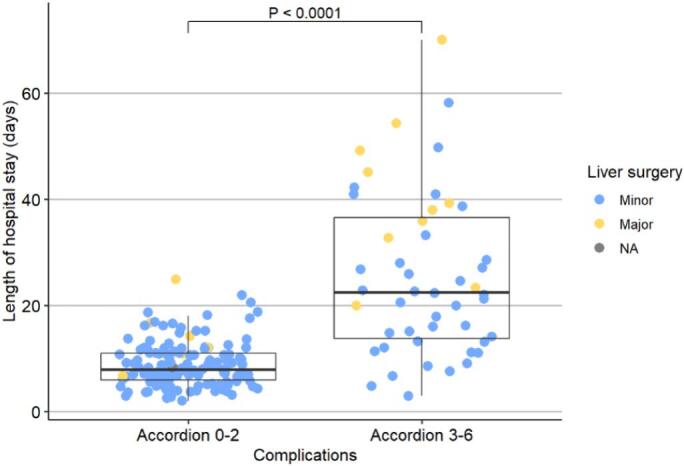
Length of hospital stay following simultaneous resection of colorectal cancer and liver metastases, stratified by postoperative complication severity (Accordion Severity Grading System) and extent of liver resection (minor vs. major). ASGS 0–2: median 8 (6–11) days vs. ASGS ≥3: median 23 (13.3–37.5) days, (p < 0.001). Minor liver resections: median 9 (6–13) days vs. major liver resections: median 24 (11.8–40.5), p < 0.001.

Median hospital stay was 9 days (IQR 7–15). Patients with minor complications (ASGS 0–2) had a median hospital stay of 8 days (ICR 6–11) compared to median 22.5 days (IQR 13.3–37.5) among those with ASGS ≥3 complications (p < 0.001, Fig. 1). Patients with rectal cancer had a shorter hospital stay (Table 2). The 30- and 90-day mortality rates in the cohort were 0.5 % (n = 1) and 2.1 % (n = 4), respectively, whereas in-hospital mortality rate was 1.0 % (n = 2). One died of multiorgan failure secondary to anastomotic leakage, and one from an infected bilioma, atrial fibrillation, and subsequent multiorgan failure (Supplemental Table 2).

### Overall survival

**Fig. 2:**
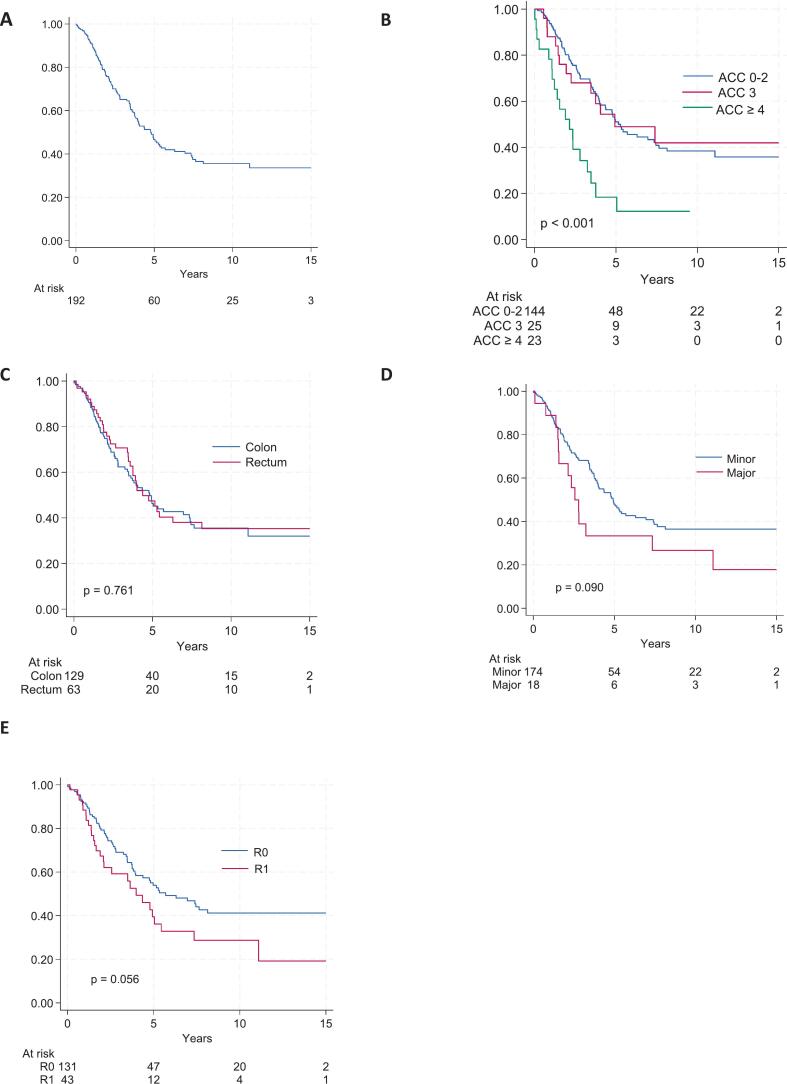
Overall survival following simultaneous resection for colorectal liver metastases 2005–2022 (n = 192) stratified by: (A) all included patients; (B) postoperative complication severity based on the Accordion Severity Grading System (ASGS); (C) primary tumour location (colon vs. rectum); (D) extent of liver resection (minor vs. major); and (E)resection margin status.

At final follow-up, 87 patients (45.1 %) were alive with a median follow-up of 6.7 years (IQR 2.7–10.2). The median, five-year and ten-year OS were 4.8 years, 46.7 %, and 35.6 %, respectively (Fig. 2A). Survival data was missing in one patient. Severe complications were significantly associated with inferior survival, with a median 5.2 years for ASGS 0–2, 4.9 years for ASGS 3; and 2.1 years for ASGS ≥4, K-M curves (Fig. 2B), log rank p < 0.001. Primary tumour location (colon vs. rectum) did not affect OS, K-M curves (Fig. 2C), log-rank p = 0.761.

**Table 2:** Perioperative outcomes in patients undergoing simultaneous resection for colorectal cancer and synchronous liver metastases 2005–2022, n = 193.

	Colon, n (%) n = 130	Rectal, n (%) n = 63	Total, n (%) n = 193	p
Op.time⁎⁎ (min)	244 (198–327)	278 (224–350)	260 (202−330)	0.052^4^
Minor^2^ LR	113 (86.9)	62 (98.4)	175 (90.7)	0.010^1^
Major^3^ LR	17 (13.1)	1 (1.6)	18 (9.3)	
Mini-invasive	23 (17.7)	14 (22.2)	37 (19.2)	0.453^1^
CRC stage				
pT0–1	2 (1.6)	4 (6.3)	5 (2.5)	0.039
pT2	8 (6.2)	6 (9.5)	14 (7.3)	
pT3	75 (57.7)	42 (66.7)	117 (60.6)	
pT4	41 (31.5)	9 (14.3)	50 (25.9)	
pTx	4 (3.1)	2 (3.2)	6 (3.1)	
pN0	39 (30.0)	19 (30.2)	58 (30.1)	0.885^1^
pN1	61 (46.9)	26 (41.3)	87 (45.1)	
pN2	25 (19.2)	14 (22.2)	39 (20.2)	
pN3	2 (1.5)	2 (3.2)	4 (2.1)	
Nx	3 (2.3)	2 (3.2)	5 (2.6)	
Resection margin				0.006^1^
R0	80 (69.0)	52 (88.1)	132 (75.4)	
R1	36 (31.0)	7 (11.9)	43 (24.6)	
ASGS				0.888^1^
0–1	65 (50.0)	33 (52.4)	98 (50.8)	
2	29 (22.3)	18 (28,6)	47 (24.4)	
3	18 (13.8)	7 (11.1)	25 (13.0)	
4	6 (4.6)	3 (4.8)	9 (4.7)	
5	10 (7.7)	2 (3.2)	12 (6.2)	
6	2 (1.5)	0 (0)	2 (1.0)	
Anastomotic leak.	6 (4.6)	3 (4.8)	9 (4.7)	0.964^1^
Hosp. stay (days)⁎⁎	13.8 (11, 7–16)	11.7 (8, 6–12)	13.1 (9, 7–15)	0.091^4^

1) Pearson Χ^2^, 2) Including wedge, atypical, segmentectomies and bi-segmentectomies, 3) Right or left hemi-hepatectomy, 4) non-parametric test (independent median samples test), 5) TN stage based on resection specimen, 6) Resection margin (R1 < 1 mm). ASGS: Accordion Severity Grading System.

⁎⁎ Median (IQR).

**Table 3:** Univariable and multivariable Poisson regression analyses of risk factors for developing severe postoperative complications (ASGS ≥3) following simultaneous resection of synchronous liver metastases (n = 193).

Variable	Univariable			Multivariable		
	RR	95 % CI	p	RR	95 % CI	p
Age/10	1.03	(0.83, 1.28)	0.804	1.07	(0.86, 1.34)	0.543
Sex, male	1.15	(0.70, 1.88)	0.587	1.24	(0.76, 2.05)	0.391
Rectum primary⁎	0.69	(0.39, 1.23)	0.206	0.76	(0.42, 1.40)	0.381
Preop. chemo.	1.30	(0.79, 2.13)	0.301	1.22	(0.74, 2.00)	0.429
Major liver resection	2.56	(1.55, 4.22)	<0.001	2.40	(1.37, 4.17)	0.002
ASA score (1–4)	1.05	(0.72, 1.51)	0.815	0.99	(0.69, 1.42)	0.942

RR: Risk ratio; CI: Confidence interval; ASA: American Society of Anaesthesiologists (ASA) physical status classification system.

⁎ Only one rectum cancer had a major hepatectomy.

Median OS following major and minor liver resections were 2.6 years and 4.9 years, respectively, K-M curves (Fig. 2D), log rank p = 0.09. Positive resection margins (R1) were present in 43 (22.4 %) patients, with a trend toward inferior survival (median OS 4.0 vs. 5.7 years; K-M curves (Fig. 2E), log rank p = 0.056). Increasing T or N stage of the primary tumour did not influence OS. In a multivariable Cox regression, the number of liver metastases and the presence of severe complications (ASGS ≥4) were independent predictors of inferior OS (p < 0.05; Table 4).

**Table 4:** COX multivariable regression analysis of overall survival in patients undergoing simultaneous resection for synchronous liver metastases 2005–2022, n = 193.

Variable	Univariable					Multivariable		
		n	HR	95 % CI%	p	HR	95 % CI%	p
Sex	Female	91	1.00	Ref	0.815	1.00	Ref	0.408
	Male	102	0.96	(0.65, 1.4)		0.83	(0.54, 1.38)	
Age/10		193	1.03	(0.88, 1.21)	0.001	1.14	(0.95, 1,38)	0.154
No. mets	1–25	193	1.08	1.02, 1.15	0.007	1.10	(1.03, 1.18)	0.004
Resection	Minor	175	1.00	Ref	0.093	1.00	Ref	0.335
	Major	18	1.62	(0.92, 2.85)		1.36	(0.73, 2.51)	
ASGS	0–2	145	1.00	Ref	0.001	1.00	Ref	0.002
	3	25	1.01	(0.55, 1.81)		0.95	(0.50, 1.81)	
	≥4	23	2.88	(1.73, 4.80)		2.90	(1.59, 5.29)	
Res. margin⁎⁎	R0	131	1.00	Ref	0.058	1.00	Ref	0.334
	R1	43	1.55	(0.99, 2.42)		1.25	(0.79, 1.98)	

HR: Hazard ratio; CI: confidence interval; ASGS: Accordion Severity Grading System.

⁎⁎ Resection margin unknown in 19 patients.

## Discussion

The main finding of this study is that simultaneous resection of CRC and CRLM was a safe approach in selected patients, particularly when minor liver resections were performed. Importantly, the location of the primary tumour (colon or rectum) did not significantly influence postoperative morbidity or mortality, although it must be observed that rectal location was almost exclusively associated with minor liver resections.

Historically, the conventional approach involved initial resection of the primary tumour, followed by an observational period of 3–6 months, and subsequent liver resection in patients without progression beyond resectability [26]. From the early 2000s, the “liver-first” approach gained traction, particularly among patients with extensive liver tumour burden and asymptomatic primary tumour [27,28]. The popularity of simultaneous resection has increased in recent years due to multiple envisioned advantages, including the potential for one-step tumour eradication and reduction in total hospital stay.

In this study, we observed a major postoperative complication rate (ASGS ≥3) of 25 %, with 30- and 90-day mortality rates of 0.5 % and 2.1 %, respectively. These outcomes are in-line with those reported in the literature [5,29,30]. Notably, patients undergoing major liver resections experienced disproportionately higher rates of adverse events, a trend that aligns with findings from other publications. An expert panel in 2015 recommended simultaneous resection only for carefully selected patients undergoing minor liver resections [31]. Bogach et al. reported increased major complication rates in patients undergoing major liver resections within a simultaneous setting (OR 1.94; p = 0.011), as well as a higher 90-day mortality rate and an inferior OS [5,8]. Similarly, Reedy et al. demonstrated increased morbidity and mortality associated with major hepatectomies following simultaneous resections [8]. Conversely, no significant differences were noted between minor liver resections and staged approaches [8]. Furthermore, Sijberden et al. found that major liver resections were associated with increased intraoperative transfusions, higher complication rates, and prolonged hospitalization compared to minor simultaneous liver resections [32]. Likewise, Yutaka et al. also observed that patients with a higher hepatic tumour burden were at greater risk of complications when undergoing simultaneous vs. staged resection [33]. In a recent published study Rashid et al. (2025) detected that the risk for textbook outcome in liver surgery (TOLS) was lower following simultaneous resection compared to staged resections. The incidence of TOLS were also lower following extensive surgery (high risk colectomy and major liver resections) [10]. A common limitation across these studies is the small sample size, and the fact that most compare minor simultaneous resections to major staged procedures, thus failing to directly address the core question of staged versus simultaneous surgery in comparable patient cohorts. In the present study, the increased proportion of severe complications was primarily driven by the complexity of the primary tumour resections, which is not necessarily avoidable by opting against simultaneous procedures.

The role of rectal cancer surgery combined with liver resection has been debated and evaluated [9]. Our findings indicate that patients with rectal cancer selected to simultaneous resection were generally younger, had lower ASA scores, very few major liver resections, and a lower incidence of positive surgical margins compared to those with colon cancer, potentially contributing to lower complication rates, shorter length of hospital stay, and improved outcomes. Notably, neoadjuvant chemotherapy did not adversely affect postoperative outcomes, consistent with previous findings [34]. Anastomotic leakage was seen in 4.7 % following simultaneous surgery. This is equivalent to a systematic review in patients undergoing colorectal surgery only [35]. In a propensity scored matched study Guerra et al. (2021) found an insignificant difference in anastomotic leakage comparing simultaneous resections (9.6 %) vs. colorectal resection alone (6.3 %) [36].

The 5- and 10-year OS rates were 46.7 % and 35.6 %, respectively, and were comparable to previously published results [37]. Variability in long-term survival rates across different studies is likely attributable to differing inclusion criteria for simultaneous resections. In a randomized controlled study by Boudjema et al. simultaneous resection was associated with superior survival and comparable complication rates relative to staged resection [38]. Vallance et al. similarly reported that all three treatment strategies (primary-first, liver-first, and simultaneous) achieved comparable long-term outcomes [39].

In our study, major liver resections were associated with a non-significant trend toward lower survival, which may reflect either higher incidence of complications or more advanced tumour burden. This is consistent with findings from Correa-Gallego et al., who reported a correlation between perioperative morbidity and poorer cancer-specific outcomes in patients undergoing major liver surgery [40]. Retrospective studies using propensity score matching have attempted to clarify any survival advantage of different surgical strategies. For instance, Sutton et al. reported poorer outcomes with the liver-first approach, raising concerns about the oncologic risk of leaving the primary tumour untreated [41]. Conversely, the LiverMetSurvey registry, analysing over 7000 patients, demonstrated a survival benefit for the liver-first strategy in advanced cases with multiple bilobar metastases, while outcomes for solitary or unilobar metastases were similar across all approaches [42]. A recent meta-analysis of 46 observational studies involving more than 20,000 patients found that simultaneous resections were associated with shorter hospital stays, reduced blood loss, and improved tumour clearance compared to staged approaches [43].

Positive resection margins demonstrated a trend toward reduced OS in a univariate analysis. The prognostic significance of R1 resections remains controversial, with ongoing debate as to whether positive margins represent an independent risk factor or merely a surrogate for aggressive tumour biology [[44], [45], [46]].

The study has several limitations that should be acknowledged. First, the study has a retrospective design and has no control group, thus precluding any comparison to alternative treatment strategies, e.g., liver first. The study period spanned nearly two decades, during which significant advancements occurred in imaging, surgical techniques, and oncological treatment. We also lack comparable national data from complications, length of hospital stays, and OS following staged resection during the same period. Furthermore, although the inclusion of all HPB units in Norway strengthens the generalizability of the findings, the difference in surgical practice of simultaneous resections and MIS may influence the interpretations of the results. Notably, the largest HPB-unit has the lowest number of simultaneous resections – primarily due to internal organisational structures rather than differing in sentiments. Additionally, unmeasured confounding variables, such as tumour biology, genetic markers, and surgeon experience may have influenced both treatment decisions and outcomes. Finally, although we stratified outcomes by tumour location and extent of liver resection, the relatively small number of patients undergoing major resections may limit the statistical power to detect clinically significant differences in survival outcomes.

## Conclusions

Major liver resections combined with colorectal surgery were associated with increased morbidity and a trend toward inferior survival outcomes. Both rectal and colon cancers can be managed with simultaneous surgery in the context of minor liver resections. These findings underscore the importance of careful patient selection, surgical planning, and multidisciplinary decision-making. However, a head-to-head comparison between staged and simultaneous resections is needed to address key clinical questions.

The following are the supplementary data related to this article.

Supplemental Table 1 Simultaneous resections for CRLM 2005–2022 according to the five HPB centres and their catchment areas in Norway. Supplemental Table 1

Supplemental Table 2 Complication (ASGS ≥4) descriptions in 23 patients. Supplemental Table 2

## CRediT authorship contribution statement

**J.H. Angelsen:** Investigation, Formal analysis, Writing – review & editing, Project administration, Writing – original draft, Methodology, Conceptualization, Software. **S. Yaqub:** Conceptualization, Writing – review & editing, Methodology, Data curation. **T.A. Hegvik:** Data curation, Formal analysis, Writing – review & editing, Conceptualization, Methodology. **L.S. Nymo:** Data curation, Conceptualization, Writing – review & editing. **T. Veen:** Data curation, Writing – review & editing, Conceptualization. **V.J. Dagenborg:** Writing – review & editing, Data curation, Conceptualization. **E.A. Bringeland:** Data curation, Writing – review & editing, Conceptualization, Methodology.

## Ethics approval

The study was approved by the Regional Committees for Medical and Health Research Ethics, Western region of Norway, 2023 (REC number: 198367) and Data Protection Officer (Haukeland University Hospital, PVO number: 4143). An information letter has been sent to surviving patients with passive informed consent.

## Funding sources

This research did not receive any specific grant from funding agencies in the public, commercial or not-for-profit sectors.

## Declaration of competing interest

Hegvik (co-author #3) owns stocks in Grail, a biotech company that primarily works with cancer screening tests, and Camurus, a pharmaceutical company that works with drugs used in treatment of several diseases. The remaining authors declare they have no commercial or financial conflicts of interest to report.
