# Supplementary material for: Outcomes of simultaneous resection for colorectal liver metastases: A nationwide cohort study (2005–2022)
Source: Surg Open Sci. 2025 Aug 5;27:113–9. doi: 10.1016/j.sopen.2025.07.008 (PMC12538211; doi:10.1016/j.sopen.2025.07.008)
Supplement: Supplemental Table 1 — Simultaneous resections for CRLM 2005–2022 according to the five HPB centres and their catchment areas in Norway. [file mmc1.docx]

**Supplemental table 1: Simultaneous resections for CRLM 2005-2022 according to the five HPB centres and their catchment areas in Norway.**

| **Region (hospital)** | **Simultaneous resections** | **Catchment area** | **Resections/100 000** |
| --- | --- | --- | --- |
| **North (Tromsø)** | 32 | 500 000 | 6.4 |
| **Central (Trondheim)** | 62 | 650 000 | 9.5 |
| **West (Bergen)** | 71 | 700 000 | 10.1 |
| **West (Stavanger)** | 16 | 370 000 | 4.3 |
| **South-East (Oslo)*** | 12 | 3 000 000 | 0.4 |

*Disparity in numbers compared to the remaining health regions in Norway is contingent on organisational structures and not differing sentiments on treatment strategies as the CRC surgery and HPB surgery may belocated at different hospitals
