# Supplementary material for: Outcomes of simultaneous resection for colorectal liver metastases: A nationwide cohort study (2005–2022)
Source: Surg Open Sci. 2025 Aug 5;27:113–9. doi: 10.1016/j.sopen.2025.07.008 (PMC12538211; doi:10.1016/j.sopen.2025.07.008)
Supplement: Supplemental Table 2 — Complication (ASGS ≥4) descriptions in 23 patients. [file mmc2.docx]

**Supplemental table 2: Complication (ASGS ≥4) descriptions in 23 patients**

| **No.** | **ASA** | **ASGS** | **Primary** | **Liver** | **Description of complications** |
| --- | --- | --- | --- | --- | --- |
| 1 | 2 | 4 | Rectum | Minor | Anastomotic leakage – reoperation |
| 2 | 3 | 4 | Colon | Minor | Anastomotic leakage – reoperation |
| 3 | 2 | 4 | Rectum | Minor | Anastomotic leakage – reoperation |
| 4 | 2 | 4 | Colon | Minor | Anastomotic leakage – reoperation |
| 5 | 2 | 4 | Rectal | Minor | Anastomotic leakage – reoperation |
| 6 | 2 | 4 | Colon | Major | Pleural empyema – reoperation (thoracotomy) |
| 7 | 2 | 4 | Colon | Minor | Mechanical bowel obstruction - reoperation |
| 8 | 3 | 4 | Colon | Minor | Wound dehiscence – reoperation. Electro conversion of atrial fibrillation under GA. |
| 9 | 3 | 4 | Colon | Minor | Perineal percutaneous drainage of pelvic abcsess under general anestesia |
| 10 | 2 | 5 | Colon | Minor | Cardiac and respiratory failure - ICU treatment |
| 11 | 2 | 5 | Colon | Minor | Atrial fibrillation, pneumonia, abscess - percutaneous drainage and ICU treatment |
| 12 | 3 | 5 | Colon | Minor | Duodenal perforation and iatrogenic small intestinal injury – reoperation and ICU treatment |
| 13 | 2 | 5 | Rectum | Major | Mechanical bowel obstruction, aspiration pneumonia and cardiac arrest, multiorgan failure - reoperation and ICU treatment |
| 14 | 3 | 5 | Colon | Minor | Anastomotic leakage and multi-organ failure – reoperation and ICU treatment |
| 15 | 2 | 5 | Colon | Major | Respiratory failure - non-invasive ventilation -ICU treatment |
| 16 | 2 | 5 | Colon | Major | Biliary leakage, multiorgan failure – reoperation and ICU treatment |
| 17 | 3 | 5 | Colon | Minor | Myocardial infarction, pulmonary embolism |
| 18 | 2 | 5 | Rectum | Minor | Respiratory failure, atelectasis, NIV |
| 19 | 2 | 5 | Colon | Major | Anastomostic leakage – reoperation, ICU treatment |
| 20 | 3 | 5 | Colon | Major | Anastomotic leakage, sepsis and multiorgan failure – Reoperation and ICU |
| 21 | 3 | 5 | Colon | Minor | Respiratory failure, hypotension – ICU treatment |
| 22 | 4 | 6 | Colon | Minor | Anastomotic leakage – reoperation – deceased |
| 23 | 4 | 6 | Colon | Major | Infected bilioma, sepsis, atrial fibrillation, multiorgan failure – deceased. |

ICU – Intensive Care Unit

ASGS – Accordion Severity Grading System

ASA - American Society of Anaesthesiologists physical status classification.
